# Supplementary material for: Surface structure determines dynamic wetting
Source: Sci Rep. 2015 Feb 16;5:8474. doi: 10.1038/srep08474 (PMC4329571; doi:10.1038/srep08474)
Supplement: Supplementary Information [file srep08474-s1.pdf]

Surface structure determines dynamic wetting

Jiayu Wang[1], Minh Do-Quang[2], James J. Cannon[1], Feng Yue[1], Yuji Suzuki[1],
Gustav Amberg[2], Junichiro Shiomi[1,3]

[1]Department of Mechanical Engineering, The University of Tokyo, Bunkyo, Tokyo, Japan

[2]Department of Mechanics, Linné Flow Centre, The Royal Institute of Technology, Stockholm, Sweden

[3]CREST, Japan Science and Technology Agency, Tokyo, Japan

1. Fabrication of microstructured surfaces

Cuboid pillars of micrometer size were fabricated by a conventional microfabrication method, which includes three main processes: electron beam lithography, non-Bosch etching and surface cleaning (Fig. S1).

1.1. Electron beam lithography

Electron beam (EB) lithography has been commonly used to define microscale patterns [S1], and this is the approach we took to directly draw patterns on electron-beam resist on the 4-inch silicon wafer (100). First of all, the native silicon dioxide layer on the surface was removed using HF (5 wt%), followed by dehydration at 180 °C for 10 minutes and spin-coating of a 400 nm-thick ZEP-520A resist layer at 4000 rpm for 60 seconds. After being soft baked at 180 °C for 15 minutes, the silicon wafer covered with ZEP-520A resist was exposed with an EB lithography system (F5112+VD01, ADVANTEST), in which the exposure dose was set to be 112 $\mu C/cm^2$. The wafer was then immersed into ZED-N50 solution for 70 seconds for development and then moved to ZMD-B for cleaning. It was confirmed through observation that the resist patterns clearly emerged on the silicon surface. The process of EB drawing is outlined in Fig. S1(a).

1.2 Etching process

Plasma dry etching is often employed for the fabrication of microscale pillars [S2]. Since the Bosch process produces scalloping and polymer surfaces on the sidewalls, a non-Bosch process [S3] was developed as a suitable method to yield vertical sidewalls for the current work. Through etching, the Si pillars with a desired height (h=1.6 μm) were manufactured in an inductively coupled plasma (ICP) etching system (MUC-21, Sumitomo Precision Products Co., Ltd.). The adopted parameters were: gas rate SF_6:O_2=50:50 sccm, pressure=3 Pa, platen power=10 W, coil power=1200 W, etching rate=1.8 μm/min, and etching time=50s. The sketch of EB drawing is shown in Fig. S1(b).

1.3 Sample cleaning

Sample cleaning [Fig. S1(c)] was conducted in order to obtain microstructures with homogeneous chemical properties. Initially, oxygen plasma was employed to remove the residual resist in a reactive-ion etching (RIE) system (O_2 flow rate: 30 sccm, pressure: 26 Pa, RF power: 100 W, etching time: 15 min, Model: RIE-10NR, SAMCO). It was found that the surface was superhydrophilic (<5°) right after the oxygen plasma treatment, and became less hydrophilic as time elapsed, in keeping with recently published results [S4]. To achieve a stable partial-wetting surface, the microstructured sample was first immerged into HF solution (5 wt%) and then exposed in air in a clean booth for three days so that the surface was saturated with native oxide layer. As a result of all the process, we obtain microstructured surface [Fig. S1 (d)] with stable and uniform chemistry.

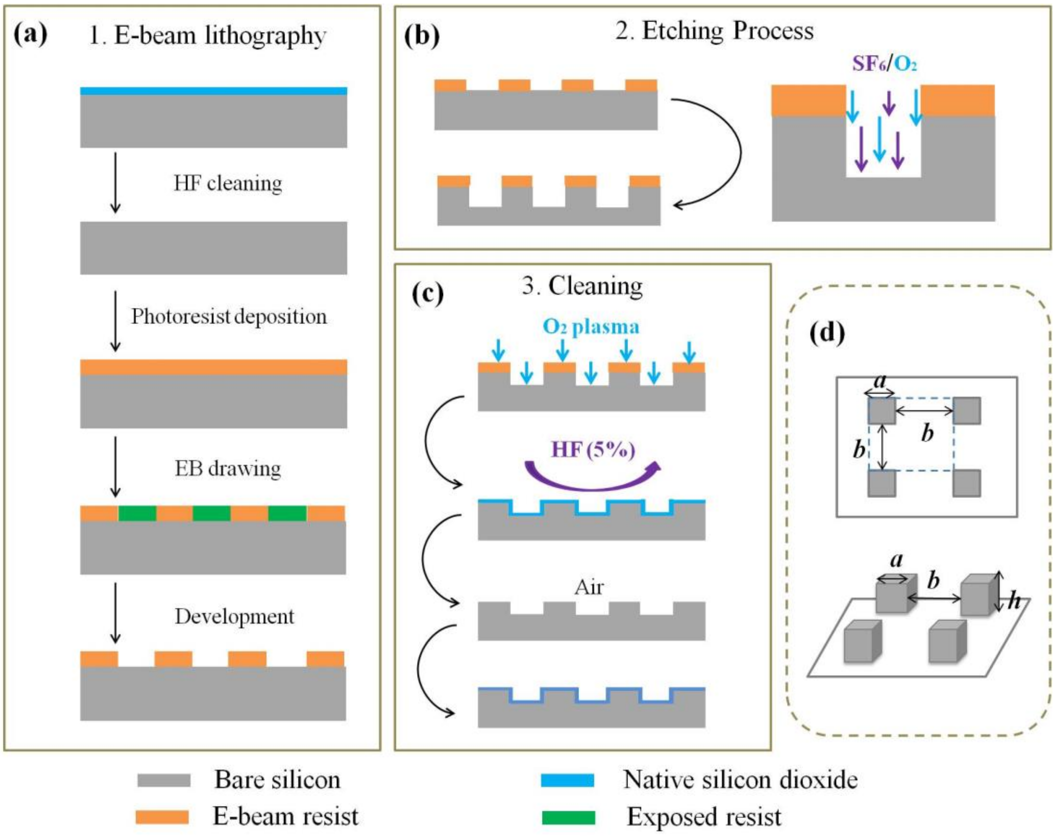

Figure S1: The microfabrication process: (a) Electron beam lithography, (b) Non-Bosch etching, and (c) cleaning process. A schematic of the microstructures is shown in (d).

2. Selection of working liquid

Water was chosen as the base working fluid to study partial wetting. In order to achieve Wenzel-state wetting for all the microstructures used in the current study, ethanol was blended

into the water to lower the surface tension. For investigation of the influence of viscosity, glycerol was chosen as another additive for three reasons. Firstly, glycerol, water and ethanol are miscible, and thus they can be mixed in any proportion to adjust physical properties. Secondly, the surface tension of glycerol and water is similar (glycerol: 0.063 N/m, water: 0.072 N/m at 20 °C [S5]) and hence the surface tension of the mixture can be maintained nearly constant if water is replaced with the same amount of glycerol. Thirdly, the dynamic viscosity of glycerol at 20 °C is 1,412 cP [S6], while the values for water and ethanol are both about 1.0 cP [S7]. This considerable difference allows us to vary viscosity over a wide range. The droplet spreading experiments were carried out for three types of mixtures resulting in dynamic viscosities of 1.0 cP, 3.3 cP, and 12.0 cP (Row #2, Tab. S1). The contact angles of these three mixtures on flat silicon substrates were about 50° (Row #3, Tab. S1), confirming their similar surface tensions and ensuring partial wetting during the spreading process.

Table S1: Properties of the working fluids: viscosity and contact angle on the flat silicon substrate.

Type of mixture	Working liquid #1	Working liquid #2	Working liquid #3
Volume ratio	Water+Ethanol (2:1)	Glycerol+Water+Ethanol (2:3:2.1)	Glycerol+Water+Ethanol (2:1:1)
Dynamic viscosity	1.0 cP	3.3 cP	12.0 cP
Contact angle	44	45	53

3. Equilibrium contact angle

After the spreading experiments, the equilibrium contact angle θ_{eq} was measured by fitting a circular arc to the periphery of the deposited droplet (Fig. S2). The equilibrium contact angle can be calculated as $\sin\theta_{eq} = r / r_0$, where r is the equilibrium radius of the droplet on the surface and r_0 is the radius of the fitted circle. The values of the measured equilibrium contact angles of working liquid #1 are shown in Tab. S2.

In the case of a Wenzel state [S8], the equilibrium contact angle θ_{eq} after spreading on a rough solid surface obeys $\cos\theta_{eq}=s\cos\theta_{eq\_flat}$, where s is the roughness of the solid. For a microstructured surface, s>1, and thus $\theta_{eq} < \theta_{eq\_flat}$. On the contrary, in case of a Cassie state [S9], $\cos\theta_{eq}=f(\cos\theta_{eq\_flat}+1)-1$, where f is the fraction of solid/liquid interface. This gives $\theta_{eq} > \theta_{eq\_flat}$ for the microstructured substrates.

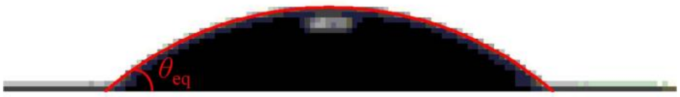

Figure S2: The circular arc fitted to the periphery of an equilibrium droplet with 1.0 cP viscosity.

Table S2: Equilibrium contact angles θ_{eq} of the water-ethanol mixture (working liquid #1) on some representative substrates.

a (μm)	*b* (μm)	θ_{eq} of working liquid #1
1	1	5.4°
2	2	26.1°
1	3	40.8°
1	4	41.4°
5	5	34.2°
Flat silicon substrate		43.8°

Since the measured equilibrium contact angles of working liquid #1 on microstructured substrates were smaller than those on the flat substrates (Tab. S2) and the surface tensions of working liquids #2 and #3 were nearly the same as working liquid #1, we judge that the Wenzel wetting is realized for all the microstructures in this study.

In Wenzel wetting, it is known that the grooves of the microstructures can wick the liquid. This has been found, through observation in late stage of wetting close to equilibrium, to become distinct in case of completely wetting [S10, P11] or partial wetting with small equilibrium contact angle [S11]. In this work, we stay within a partial wetting regime where the wicking is absent even in late stage of wetting. The absence of wicking was confirmed by observing the wetting from the top of the surface, and is consistent with the reported criterion for wicking [S13]. Note that even in the strongly wetting cases with wicking in late stage, the extent of wicking is expected to be minute in the initial stage of wetting, since the wetting is rapid.

4. Advancing and receding contact angles

Contact angle hysteresis [S15], the difference in advancing and receding contact angles, was measured by the dynamic sessile droplet method. The procedure is illustrated using the selected

snapshots in Fig. S3 taken for the case of (a, b)=(2, 2). At first, the droplet with 1.0 mm diameter was deposited on the substrate, displaying an equilibrium contact angle [Fig. S3(a)]. The droplet was then inflated by adding more liquid using a syringe pump. Liquid was dispensed little by little so that the volume and contact angle of the droplet grew gradually [Fig. S3(b)] until the contact line advanced [Fig. S3(c)]. The maximum contact angle recorded right before the contact line advances is defined as the advancing contact angle. Next, we performed the reverse process by slowly subtracting liquid from the droplet [Fig. S3(e)] until the contact line receded [Fig. S3(f)].

As shown in Fig. S4, the advancing contact angle significantly increases with the roughness parameter until it saturates for the large roughness parameters, independently of viscosity. The dependence of the advancing contact angle on roughness is in agreement with an early experiment by Johnson and Dettre [S16]. On the other hand, the dependence of receding contact angle on the roughness parameter in our experiment is much weaker than that found in Ref. 15. The difference could be explained by the dissimilar geometries of structure or different properties of liquid and solid. In fact, recent works also found different trends from Ref. 15: Pu and Severtson [S17] presented that advancing (receding) contact angle gradually increases (decreases) with increasing roughness [S17], and Chibowski and Jurak [S18] observed irregular trends of contact angles with respect to roughness [S18]. Furthermore, Nakae et. al., [S19] pointed out that the hysteresis can vary among different patterns even for the same roughness parameter [S19]. Therefore, knowledge in the advancing and receding contact angle on roughness is still insufficient to discuss

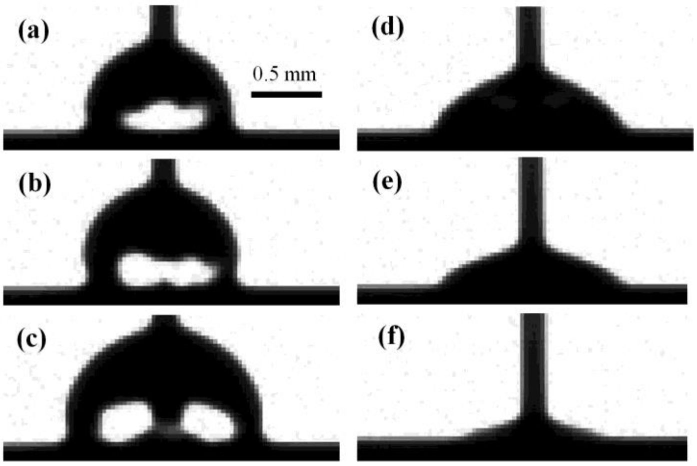

Figure S3: Snapshots of advancing and receding contact-angle measurement, for the case with (a, b)=(2, 2). (a~c) Snapshots in sequence for measurement of the advancing contact angle. (d~f) Snapshots in sequence for measurement of the receding contact angle. (b) is the selected snapshot for estimation of the advancing contact angle, while (e) is the selected snapshot for estimation of the receding contact angle.

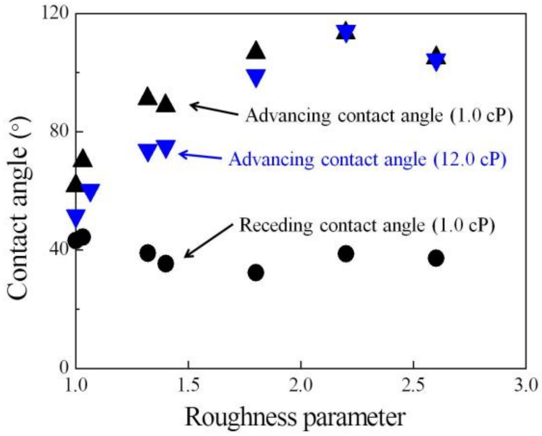

Figure S4: Advancing and receding contact angle for microstructured surfaces. The roughness parameter is defined as the total area $(a+b)^2+4ah$ of the microstructured surface divided by the projected area $(a+b)^2$. The black symbols represent cases with low viscosity, while blue symbols represent those with high viscosity. Triangles represent the advancing contact angles θ_{ad} and circles the receding contact angles θ_{re}.

detail underlying physics in terms of surface roughness. Nevertheless, we here note that the range of magnitudes of hysteresis in our experiments covers from those of macroscopically smooth [S20, S21] to larger-roughness [S16-S19] surfaces reported in the previous works.

5. Set up of spreading experiment

A set of droplet spreading experiments were conducted on all the microstructured/flat samples with the three types of mixtures described above. The experimental set up is illustrated in Fig. S5. For each trial, the substrate was placed under the thin needle (needle gauge: 33G, 90°) of which the outer diameter is 0.21 mm. The distance between the needle tip and the substrate was accurately fixed to be around 1.0 mm, which also determines the diameter of the droplet. Therefore, as soon as the droplet was extruded to be a quasi static sphere with the diameter the same as the height of the needle, the droplet came into contact with the surface and spread immediately. This rapid spreading process was captured and recorded by a high speed camera (Photron FASTCAM SA2) at 54,000 frames per second. A lamp (Photron HVC-SL) was used to provide lighting with sufficient intensity. In addition, a syringe pump (KDS 120) was used to deliver the liquid at a stable and small enough volume rate. After the experimental operations, images were processed to extract the time histories of spreading radius.

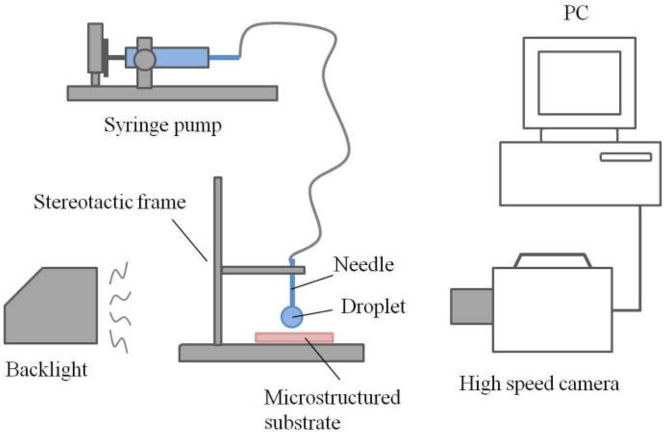

Figure S5: The sketch of spreading experiment set up.

6. Quantification of the spreading radius

From the obtained digital image [Fig. S6(a)], the boundary of the droplet was determined in two steps. Since the whole substrate was always dark and the background of the droplet was bright, the contact surface between the working liquid and the substrate could be easily probed. In processing the image, the bottom of the image was cut exactly at the contact surface and the redundant substrate was eliminated. For the periphery of the droplet, if the grey level of a pixel was lower than 60, the spot was regarded as a part of the droplet rather than background or shadow. We assigned the droplet part with gray=0, while the background or shadow was assigned gray=255, yielding the digitalized image in Fig. S6(b). The two red lines are marked to show the consistency of spreading radius between Fig. S6 (a) and (b), and to highlight accurate identification of the edge of the contact line. The spreading radius was calculated by comparing the pixel numbers of the contact line to that of the known needle width.

7. Extraction of the normalized spreading rate

The average spreading rates were calculated based on the histories of spreading radius. The spreading radius was observed to develop linearly with time over a significant portion of each measurement, and therefore a linear least-squares fit was suitable to obtain the spreading rate. The spreading radius range over which this linear fit was applied was fixed for each value of viscosity, and was chosen to be appropriate for the range of radii observed as the roughness was varied.

Spreading radius histories were recorded before deformation of the upper-half of the droplet. The histories of spreading radius for all the microstructured and flat surfaces are plotted in Fig. S7(a). The spreading radius was observed to develop linearly with time over a significant portion

of each measurement, and therefore a linear least-squares fit was suitable to obtain the spreading rate. For instance, in a low-viscosity case the histories of spreading radius in the red dashed frame (between r=0.13~0.225 mm) were chosen. The obtained average spreading rates for all the viscosities and surface structures are shown in Fig. S7(b).

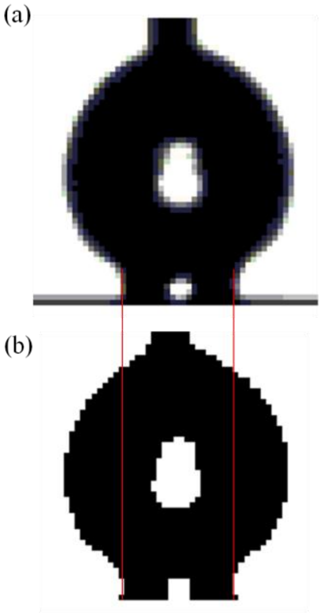

Figure S6: Example images highlighting the method used to extract the spreading radius from one snapshot. Here the low-viscosity (1.0 cP) droplet is spreading on flat substrate at t=0.241 ms. (a) The original photograph taken by high-speed camera. (b) Digitized image after processing.

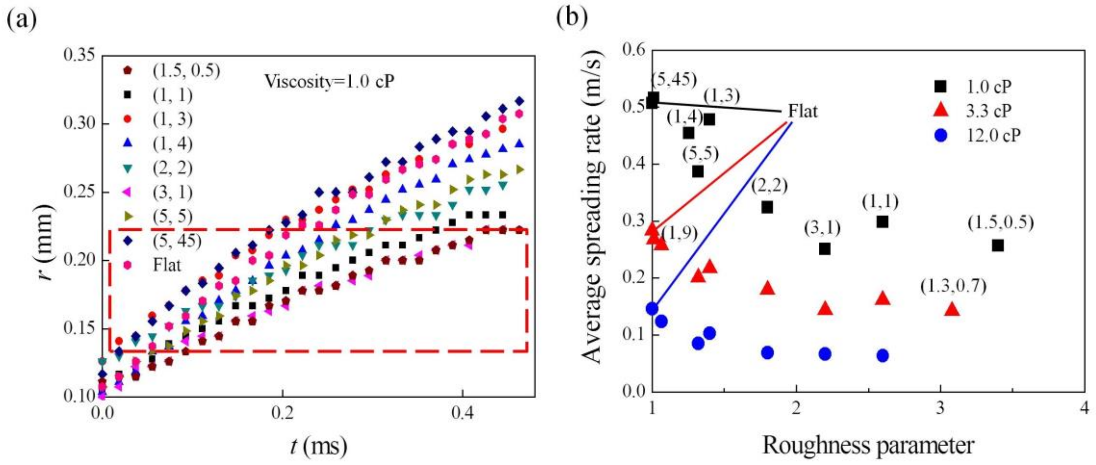

Figure S7: (a) Time histories of spreading radius for all the trials with 1.0 cP liquid. The r vs t curves in red dished frame were chosen for the calculation of average spreading rates. (b). The plots of average spreading rates with roughness parameter for three-viscosity cases.

8. Numerical simulations

8.1 Governing equations

To understand the experimental results, corresponding numerical simulation based on free energy theory was performed [S23]. To avoid violating the no-slip condition, this method describes the movement of the contact line by a diffusive interfacial flux. The governing equations of this method; a coupled equation of Cahn-Hilliard and Navier-Stokes [S24] equations for time dependent incompressible flow of a binary system; are given as below:

Cahn-Hilliard equation:

$$\frac{\mathrm{D}C}{\mathrm{D}t}=\frac{1}{\mathrm{Pe}}\nabla^2\left[\Psi'(C)-\mathrm{Cn}^2\nabla^2 C\right],\quad \text{in } \Omega_f \tag{S1}$$

Here C denotes the continuous phase field variable, which takes the value -1 in the gas and 1 in the liquid. Pe= UL/D is the Peclet number, expressing the ratio between advection and diffusion, where U and L are the characteristic velocity and length scale, and D is the bulk diffusivity. The Cahn number Cn=ε/L expresses the ratio between the interface width (ε) and the characteristic length scale. $\Psi=(C+1)^2(C-1)^2/4$ is a double-well function [S25] with two stable minima that express the two equilibrium concentrations for gas $C=-1$ and liquid $C=1$.

The Navier-Stokes equations:

$$\rho(C)\frac{\mathrm{D}\mathbf{u}}{\mathrm{D}t}=-\frac{1}{\mathrm{Re}}\nabla p+\frac{1}{\mathrm{Re}}\nabla\cdot\tau-\frac{1}{\mathrm{CnWe}}C\nabla\phi,\quad \text{in } \Omega_f \tag{S2}$$

$$\nabla\cdot\mathbf{u}=\mathbf{0} \tag{S3}$$

Here the Reynolds number Re = $\rho_l UL/\mu_l$ describes the relative importance between the inertial and viscous force in the flow, the Weber number (We) expresses the ratio between the inertia and surface tension and τ is the Newtonian viscous stress tensor, $\tau=\mu(C)\left[\nabla\mathbf{u}+(\nabla\mathbf{u})^{\mathrm{T}}\right]$. The density $\rho(C)$ and viscosity $\mu(C)$ depend on C according to $\rho(C)=0.5[(C+1)-(\rho_g/\rho_l)(C-1)]$, and $\mu(C)=0.5[(C+1)-(\mu_g/\mu_l)(C-1)]$ respectively, where subscript g denotes gas phase and l denotes liquid phase. The term $C\nabla\phi$ models the surface tension force, where $\phi=\Psi'(C)-\mathrm{Cn}^2\nabla^2 C$ is the chemical potential.

The boundary condition for the concentration on the solid surface is defined as a function of both the local equilibrium and the non-equilibrium of the wetting contact angle [S26],

$$\varepsilon\mu_{\mathrm{f}}\frac{DC}{Dt}=-\varepsilon\sigma\nabla C\cdot\mathbf{n}+\sigma_{\mathrm{lg}}\cos(\theta_{\mathrm{e}})g'(C) \tag{S4}$$

Here μ_f is interpreted as a friction factor at the contact line and the left hand side of the equation represents the non-equilibrium effects. $\cos(\theta_{\mathrm{e}})=(\sigma_{\mathrm{sg}}-\sigma_{\mathrm{sl}})/\sigma_{\mathrm{lg}}$ is the static equilibrium contact

angle, and σ_{sg}, σ_{sl} and σ_{lg} are the surface energies for the solid-gas, solid-liquid and liquid-gas interfaces, respectively. $g(C)=0.5 + 0.75C - 0.25C^3$ is a normalized function varying smoothly from 0 to 1 between gas and liquid phases.

8.2 Numerical treatment

The numerical simulations were carried out using femLego [S27], a symbolic tool for solving partial differential equations with adaptive finite element method. The partial differential equations, boundary conditions, initial conditions, and the method of solving each equation are all specified in a Maple worksheet. The Cahn–Hilliard equation is treated as a coupled system of the chemical potential ϕ and the composition C. Both chemical potential and composition equations are discretized in space using piecewise linear functions and discretized in time using a three-level backward differentiation formula (BDF2). The linear systems of ϕ and C are solved simultaneously using efficient preconditioner 2×2 blocks [S28]. The Navier–Stokes equations are discretized in space using P2/P1 finite elements and solved by a projection method for variable density first introduced by Guermond and Quartapelle [S29]. An adaptively refined and derefined mesh has been used to ensure sufficient mesh resolution along the vicinity of the interface and the surface of the microstructured substrate [Fig. S8(b)].

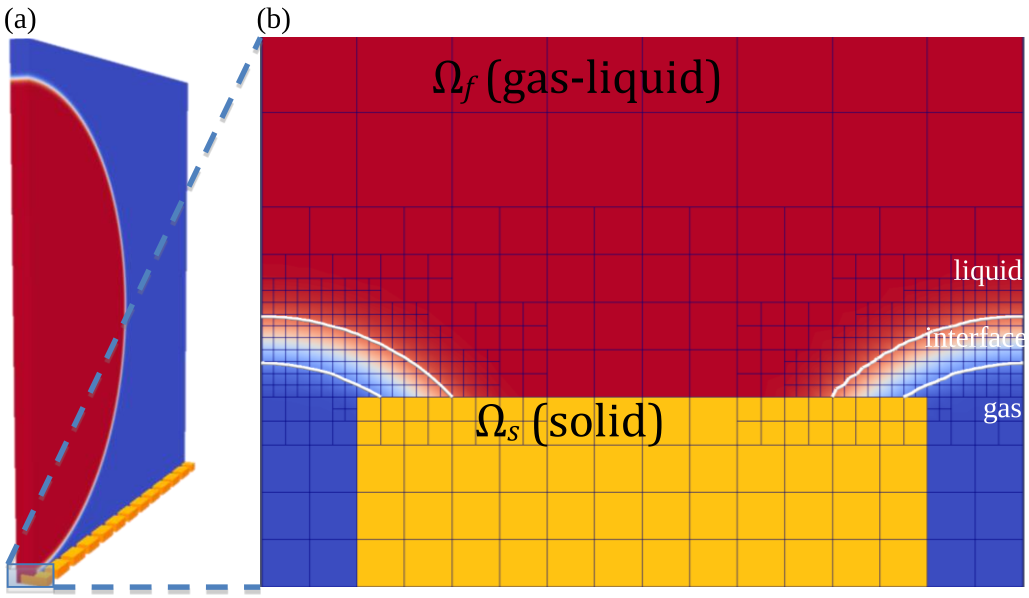

Figure S8: Three-dimension simulation domain (a) and a cross section over a groove (b).

8. 3 Set up of numerical simulations

A set of axisymmetric simulations have been performed for the full size droplet simulations. In this simulation, all the surfaces were assumed to be flat and the effect of microstructures was

incorporated through the contact line friction factor μ_f of the boundary condition (Eq. S4). On the other hand, for the detailed simulation of flow over microstructured surfaces, the simulation has been conducted on a slice in the 3D domain for a cylindrical droplet [Fig. S8(a)]. Here the slice thickness is 2(*a*+*b*), while the height and the width of the slice are 3 and 2 times the radius of the droplet, respectively. In this simulation, the solid-liquid-gas problem is defined as a coupling problem of different physics (a two-phase fluid and a rigid solid) in different parts of the domain (in Ω_f and Ω_s), as shown in Fig. S8 (b).

9. Identification of μ_f

Firstly, the value of μ_f in the axisymmetric CHNS simulations on flat surfaces was tuned iteratively to match the time histories of spreading radius to that of the experiment. Secondly, the droplet profiles at different times was visualized and the matching to that of the experiment was confirmed. The obtained values of μ_f for all the spreading measurements are listed in Tab. S3.

Table S3: The line friction coefficient μ_f (Pa s) extracted by combined analysis of experiment and numerical simulation, for each surface and viscosity.

Substrate (*a*, *b*)	μ_f (Pa s)		
	1.0 cP	3.3 cP	12.0 cP
(1.5, 0.5)	0.294	--	--
(1.3, 0.7)	--	0.978	--
(1, 1)	0.255	0.978	1.914
(1, 3)	0.106	0.456	0.957
(1, 4)	0.122	--	--
(2, 2)	0.255	0.717	1.674
(3, 1)	0.294	--	2.153
(5, 5)	0.157	0.587	1.196
(1, 9)	--	0.196	0.478
(5, 45)	0.074	0.196	--
Flat	0.090	0.196	0.478

10. Estimation of viscous dissipation

We will now proceed to estimate different possible mechanisms for this dissipation. One candidate for the energy dissipation due to the presence of the microstructure is the work of

internal viscous stresses in the complicated viscous flow around the pillars, and in the grooves between them. Assuming that a Reynolds number based on the structure length scale $Re_m=\rho U(a+b)/\mu$ is of order unity or less, the dissipation per volume of liquid is of the order $\mu(\partial u/\partial x)^2$. The dissipation associated with moving the contact line a distance $a+b$, is then $\mu(\partial u/\partial x)^2 V/l$, per length l along the contact line, with μ and V denoting the liquid viscosity and the volume of the sheared liquid. For a case with a, b and h [see Fig. 1(d)] of similar magnitudes, $\partial u/\partial x \sim U/a$, and thus, $V\sim abh$ and $l = a+b$ over one period of the pattern. The resulting expression for line dissipation is $w \sim \mu U^2\, bh/a/(a+b)$. The last geometrical factor will be of order unity for the substrates in our study, so that in fact $w \sim \mu U^2$. In equating this with the contact line dissipation as parameterized with the line friction in Eq. (1), we get $\mu_{f\_eff}U^2 \sim \mu U^2$, which would indicate that the measured μ_{f_eff} would be of the same order of magnitude as the liquid viscosity μ. This however is not the case, instead μ_{f_eff} is between forty and several hundred times larger than μ for our experiments (see Tab. S3), and we must conclude that the added viscous dissipation for the flow through the microstructures is negligible compared to the line friction dissipation.

Reference for supplemental information

[S1] Tseng, A. A., Chen, K., Chen, C. D. & Ma, K. J. Electron beam lithography in nanoscale fabrication: recent development. *IEEE T. Electron. Pack.* **26**, 141-149 (2003).

[S2] Senturia, S. D. *Microsystem Design*. (Springer, New York, 2000).

[S3] Kirikae, D., Suzuki, Y., & Kasagi, N. A silicon microcavity selective emitter with smooth surfaces for thermophotovoltaic power generation. *J. Micromech. Microeng*. **20**, 104006 (2010).

[S4] Preston, D.J., Miljkovic, N., Sack, J., Enright, R., Queeney, J. and E.N. Wang, Effect of hydrocarbon adsorption on the wettability of rare earth oxide ceramics. *Applied Physics Letters*,. **105**, 011601 (2014).

[S5] Jasper, J. J. The surface tension of pure liquid compounds. *J. Phys. Chem. Ref. Data* **1**, 841-1009 (1972).

[S6] Segur, J. B. & Oberstar, H. E. Viscosity of glycerol and its aqueous solutions. *Ind. Eng. Chem*. **43**, 2117-2120 (1951).

[S7] Khattab, I. S., Bandarkar, F., Fakhree, M. A. A. & Jouyban, A. Density, viscosity, and surface tension of water+ethanol mixtures from 293 to 323K. *Korean J. Chem. Eng*. **29**, 812-817 (2012).

[S8] Wenzel, R. N. Resistance of solid surfaces to wetting by water. *Ind. Eng. Chem.* **28**, 988-994 (1936)

[S9] Cassie, A. B. D. & Baxter, S. Wettability of porous surfaces. *Trans. Faraday Soc*. **40**, 546-551 (1944).

[S10] Lenormand, R. Liquids in porous media, *J. Phys.: Condens. Matter* **2**, SA79-SA88 (1990).

[S11] Yuan, Q. & Zhao, Y. P. Multiscale dynamic wetting of a droplet on a lyophilic pillar-arrayed surface. *J. Fluid Mech*. **716**, 171-188 (2013).

[S12] Courbin, L., Bird, J. C. & Stone, H. A. Dynamics of wetting: from inertial spreading to viscous imbibitions. *J. Phys.: Condens. Matter* **21**, 464127 (2009).

[S13] Bico, J., Tordeux, C. & Quéré, D. Rough wetting. *Europhys. Lett.* **55**, 214-220 (2001)

[S14] Eral, H. B., Mannetje, D. J. C. M. 't & Oh, J. M. Contact angle hysteresis: a review of fundamentals and applications. *Colloid. Polym. Sci*. **291**, 247-260 (2012).

[S15] De Gennes, P.G. Weting-statics and dynamics. *Rev Mod Phys* **57**, 827-863 (1985).

[S16] Johnson, R. E. & Dettre, R. H. Contact angle hysteresis, in Contact Angle, Wettability, and Adhesion, Advances in Chemistry Series **43**, 112 (1964).

[S17] Pu, G. & Severtson, S. J. Stabilizing contact angle hysteresis of paraffin wax surfaces with nanoclay. *Colloid Polym Sci* **291**, 383-389 (2013).

[S18] Chibowski, E. & Jurak, M. Comparison of contact angle hysteresis of different probe liquids on the same solid surface. *Colloid Polym Sci* **291**, 391-399 (2013).

[S19] Nakae, H., Inui, R., Hirata, Y. & Saito, H. Effects of surface roughness on wettability, *Acta Mater* **46**, 2313-2318 (1998).

[S20] Extrand, C. W. & Kumagai, Y. An experimental study of contact angle hysteresis. *J. Colloid Interface Sci***.** **191**, 378-383 (1997).

[S21] Oliver, J. P., Huh, C. & Mason, S. G. An experimental study of some effects of solid surface roughness on wetting. *Colloids Surf.* **1**, 79-104 (1980).

[S22] Meiron, T. S., Marmur A. & Saguy, I. S. Contact angle measurement on rough surface. *J. Colloid Interface Sci*. **274**, 637-644 (2004).

[S23] Carlson, A., Bellani, G. & Amberg, G. Contact line dissipation in short-time dynamic wetting. *Europhysics Lett.* **97**, 44004 (2012).

[S24] Jacqmin, D. Contact-line dynamics of a diffuse fluid interface. *J. Fluid Mech*. **402**, 57-88 (2000).

[S25] Amberg, G. Semisharp phase field method for quantitative phase change simulations. *Phys. Rev. Lett.* **91**, 265505 (2003).

[S26] Carlson, A., Do-Quang, M., & Amberg, G. Modeling of dynamic wetting far from equilibrium. *Phys. Fluids* **21**, 121701 (2009).

[S27] Do-Quang, M., Villanueva, W., Singer-Loginova, I. & Amberg, G. Parallel adaptive computation of some time-dependent materials-related microstructural problems. *Bull. Pol. Ac.: Tech.* **55**, 229-237 (2007).

[S28] Boyanova, P., Do-Quang, M. & Neytcheva, M. Efficient Preconditioners for Large Scale Binary Cahn-Hilliard Models. *Comput. Methods Appl. Math* **12**, 1–22 (2012).
[S29] Guermond, J.-L. & Quartapelle, L. A projection FEM for variable density incompressible flows. *J. Comput. Phys.* **165**, 167-188 (2000).
